# An Intra-Body Power Transfer System via Localized Capacitive Coupling

**DOI:** 10.1109/OJEMB.2026.3688340

**Published:** 2026-04-29

**Authors:** Noor Mohammed, Sunghoon Ivan Lee, Robert W. Jackson, Jeremy Gummeson

**Affiliations:** Electrical and Computer EngineeringUniversity of Massachusetts Amherst 14707 Amherst MA 01003 USA; College of Information and Computer ScienceUniversity of Massachusetts Amherst 14707 Amherst MA 01003 USA

**Keywords:** Intra-body power transfer (IBPT), localized capacitive coupling (LCC), wireless power transfer, Dickson charge pump, capacitive human body communication, wearable device

## Abstract

Intra-body power transfer (IBPT) enables batteryless wearables by using the human body as a conductive medium. This work introduces Localized Capacitive Coupling (LCC), a new IBPT technique that uses a 40 MHz RF carrier to provide power transfer without relying on external grounds or environmental infrastructure. We evaluate LCC using computational modeling and human subject experiments with ten participants across multiple short-range capacitive body channels. Laboratory measurements with an isolated electrode system show mean path gains of 44 dB to 48 dB for channel lengths of 5 to 12 cm, closely matching our computational model with deviations under 3 dB. Circuit analysis indicates that air-gap coupling capacitance, typically in the femtofarad range, dominates channel gain, highlighting the importance of short-range fringing fields. To demonstrate practical energy harvesting, we designed and evaluated multistage Dickson charge pump (DCP) receivers. A five-stage impedance-matched DCP produced 3 V DC at 13.5 $\pm$ 0.5 dBm of incident RF power across a load in the tens of megaohms. A single-stage DCP paired with a battery manager generated a regulated 1.8 V output by charging a 100 $\mu F$ capacitor from RF peak powers as low as 18.5 dBm. These results enabled a fully batteryless ring-worn motion sensor that uses an ultra-low-power accelerometer and non-volatile memory for offline activity logging, demonstrating that LCC is a practical approach for powering short-range wearable sensor networks. All hardware designs and simulation configurations will be open-sourced upon publication.

## I. Introduction

Advanced CMOS technology has enabled ultra-low-power wearable devices that integrate sensing, communication, and computational capabilities into a single platform. However, current energy supply solutions that rely on on-device batteries impose significant challenges for realizing a body sensor network composed of multiple wearable nodes. Despite notable improvements in battery energy density, supporting a network of several battery-powered devices remains impractical because of frequent recharging and battery replacement requirements [1], [2]. As a result, offline charging can interrupt continuous monitoring of an individual's health status and may lead to the loss of important physiological information [1], [2]. In addition, individuals undergoing recovery following neurological injury, orthopedic surgery, or cardiac procedures often rely on wearable systems during home exercise programs. Reducing or eliminating charging requirements may decrease device abandonment and enable more sustained physiological monitoring in such populations.

An alternative approach to powering wearable sensors is to collect energy while the devices are being worn rather than relying on a precharged source. Such systems either harvest ambient energy or wirelessly receive power from a separate transmitting device [3], [4]. Recent studies have focused on the latter, known as Intra-Body Power Transfer (IBPT), which uses the human body as a conductive medium for power delivery between wearable devices by exploiting the finite conductivity of biological tissues [1], [2], [5], [6], [7], [8], [9], [10]. This approach has emerged as a promising solution to support networks of batteryless wearable devices while requiring only one or a few battery-powered devices to supply power to the entire system.

Several technical methods have been proposed to implement IBPT, including galvanic coupling [11] and magnetic coupling techniques [12], [13]. Among them, capacitive coupling has become one of the most appealing options for wearable applications because it accommodates a wide variety of device placements and body orientations [8], [10], [14], [15], [16]. However, despite progress in capacitively coupled IBPT, substantial challenges remain in achieving a complete understanding of power transfer mechanisms, path gain, coupling capacitance, electric field distribution, and safety considerations. These challenges arise largely from two issues: (1) the need for accurate models of capacitively coupled IBPT over realistic body channels, and (2) the need for instrumentation that can reliably quantify the actual power delivered to a receiver.

**Fig. 1.**
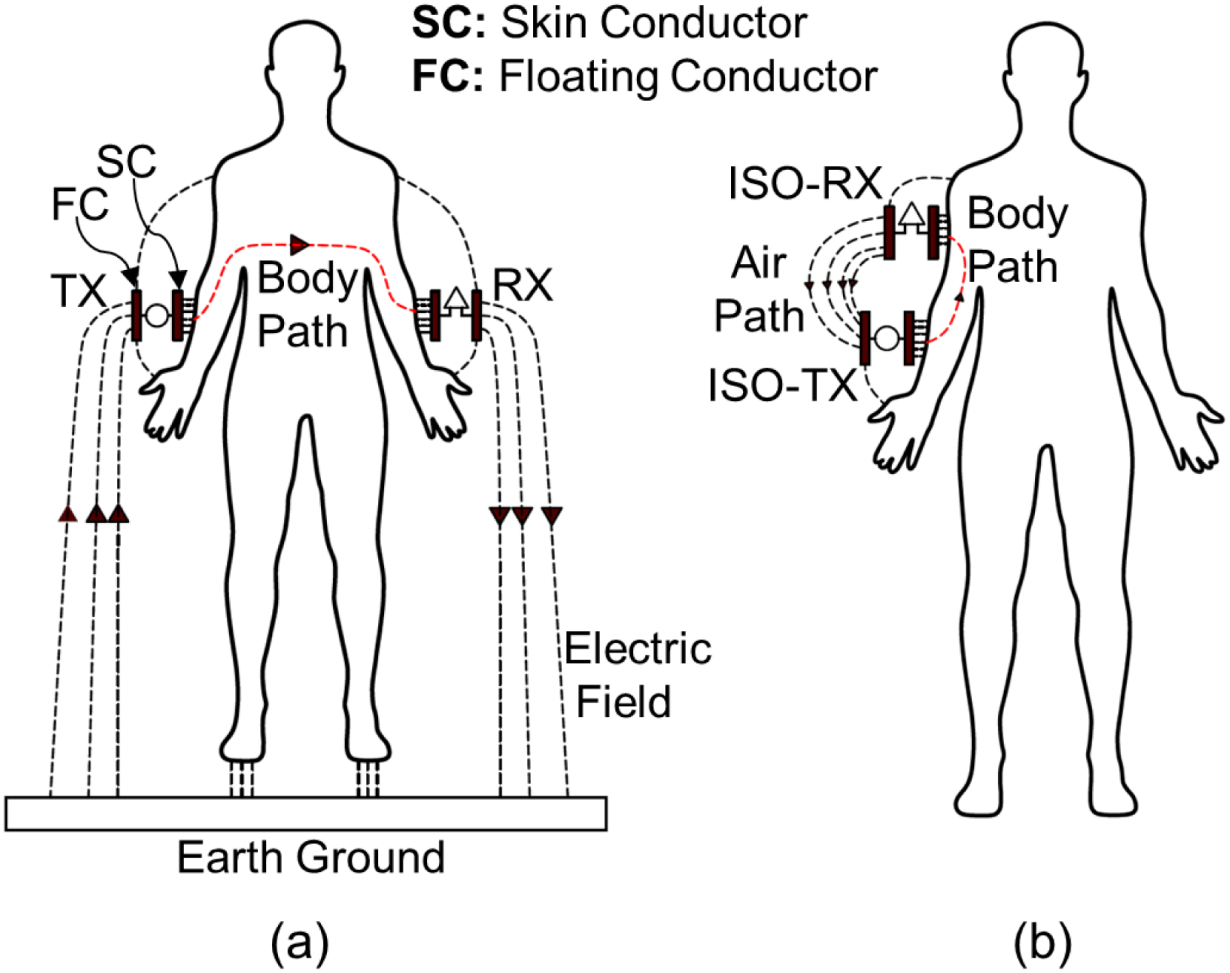
(a) Conventional earth ground coupling method of capacitive body power transfer. (b) A localized capacitve coupling power transfer system uses isolated electrode pair on the transmitter (ISO-TX) and receiver (ISO-RX) to establish body and air current paths.

A capacitively coupled IBPT system consists of a power transmitter and a receiver, each equipped with two conductive electrodes, as illustrated in Fig. 1. The body path is formed through conduction between the skin electrodes, while the air path arises from capacitive coupling between the floating electrodes. Traditional IBPT models, adapted from Intra-Body Communication [9], [17], assume long channels where coupling occurs not only between the floating electrodes but also with the surrounding environment, as shown in Fig. 1(a). These assumptions, however, do not align with IBPT's primary goal: delivering sufficient power to operate battery-free, ultra-low-power wearable devices over relatively short body channels (e.g., less than 12 cm). In such scenarios, inter-electrode coupling dominates, while environmental coupling becomes negligible, significantly affecting end-to-end power transfer modeling.

Accurate characterization of such channels requires strict ground isolation between the measurement instrument and the electrode system [10], [12]. Prior IBPT studies have often relied on benchtop equipment to measure path gain [6], [12], [18], [19], [20], [21], [22]. Although these instruments offer high measurement accuracy, they introduce a low-impedance return path through direct or indirect capacitive coupling to the floating reference. As a result, the instrument ground contributes to unintended coupling between the transmitter and receiver, leading to an overly optimistic estimate of power transfer. Reported gains can be inflated by more than an order of magnitude [2], [10], [18], [19], [23], far beyond what self-contained wearable systems can achieve. Short-channel IBPT models are especially susceptible to these artifacts because they assume minimal environmental coupling.

Several techniques have been investigated to achieve external ground isolation, including transformer-based DC-isolated baluns and optical isolation. However, neither approach provides complete isolation. Baluns can introduce unintended low-impedance paths due to inter-winding capacitance, which increases current flow through the capacitive loop [10], [20], [21], [22]. Optical isolation is effective for communication signals but has not been used to quantify energy captured by a passive charge pump [24], [25]. Some studies have used custom wearable data loggers to measure rectified DC power [9], [10], but these systems often rely on Bluetooth transmission to report data, which can introduce additional coupling paths and lead to artificially elevated gain measurements.

In this paper, we introduce an optimized model, Localized Capacitive Coupling (LCC), and its implementation for capacitively coupled IBPT over short channels. Unlike traditional approaches that maximize coupling capacitance through external earth-ground coupling [6], [8], LCC uses well-defined floating electrodes that do not rely on long-distance coupling to a large ground plane, as illustrated in Fig. 1(b). This design enables a fully self-contained power transfer system and a measurement method suitable for short body channels, while providing a simplified circuit model and realistic path gain characterization for wearable applications. Moreover, to the best of our knowledge, this work is the first to implement fully isolated power-transfer measurementsfree from the confounding factors discussed earlierthus offering more accurate power profiles for IBPT systems. The key contributions of this work include:

• We demonstrate the feasibility of the LCC system using a passive power receiver implemented within a wearable form factor. Experiments with ten human subjects show that the proposed system can deliever sufficient power to support intermittent workloads comparable to those used in RFID systems.
• We present measurements of realistic power transfer between a fully isolated transmitter and receiver using our custom-designed instrumentation. The device is miniaturized to a wearable form factor and provides full ground isolation and impedance matching. Its ability to perform in-situ power measurements eliminates stray coupling typically introduced by benchtop equipment and allows us to characterize the impact of dynamic body movements on capacitive coupling between devices.
• We validate the LCC concept through circuit modeling and finite element simulation. We use two finite element models, including a multilayer cylindrical tissue model and a full-scale commercial CAD model of a female body [26]. We also compare these models with an equivalent circuit representation to extract inter-device coupling characteristics and evaluate path gain relative to empirical measurements.
• We demonstrate a real-world application based on LCC: a batteryless wearable ring with a three-axis accelerometer. The system incorporates a single-stage charge pump, a DC-DC boost-buck converter, an ultra-low-power accelerometer, and a microcontroller, all powered entirely through IBPT.
• All hardware designs and simulation configurations will be open-sourced and made publicly available to the research community upon publication.

## II. Design Methodology

### A. Overview of Localized Capacitive Coupling (LCC)

Existing circuit models that rely on external ground coupling (i.e., Fig. 1(a)) are well suited for active communication between two battery-powered devices, where the receiver can employ high-gain amplifiers to compensate for signal attenuation. In contrast, batteryless passive receivers, similar to those used in RFID systems, cannot rely on highly attenuated signals because they lack the power required to operate amplification electronics. To address this limitation, LCC maximizes localized capacitive coupling by confining the fringing electric field between two floating electrodes with a surface area comparable to that of a modern smartwatch. This behavior, however, is only achievable when the electrodes are separated by relatively small distances (e.g., less than 12 cm). The following subsections outline the primary elements required to optimize LCC, including its hardware architecture, implementation details, and biophysical modeling.

### B. Hardware Systems

The LCC hardware platform consists of three components: 1) an electrode system, 2) a transmitter system, and 3) a power receiver system.

#### 1) Electrode System

We designed the LCC electrode system to support power transfer studies across multiple body locations, including the wrist, forearm, finger, and shank. This facilitates evaluation of several potential applications, such as batteryless ring sensors, biopatches, and smart shoe inserts. Understanding how the inter-electrode medium responds to anatomical constraints is essential for assessing power transfer performance.

**Fig. 2.**
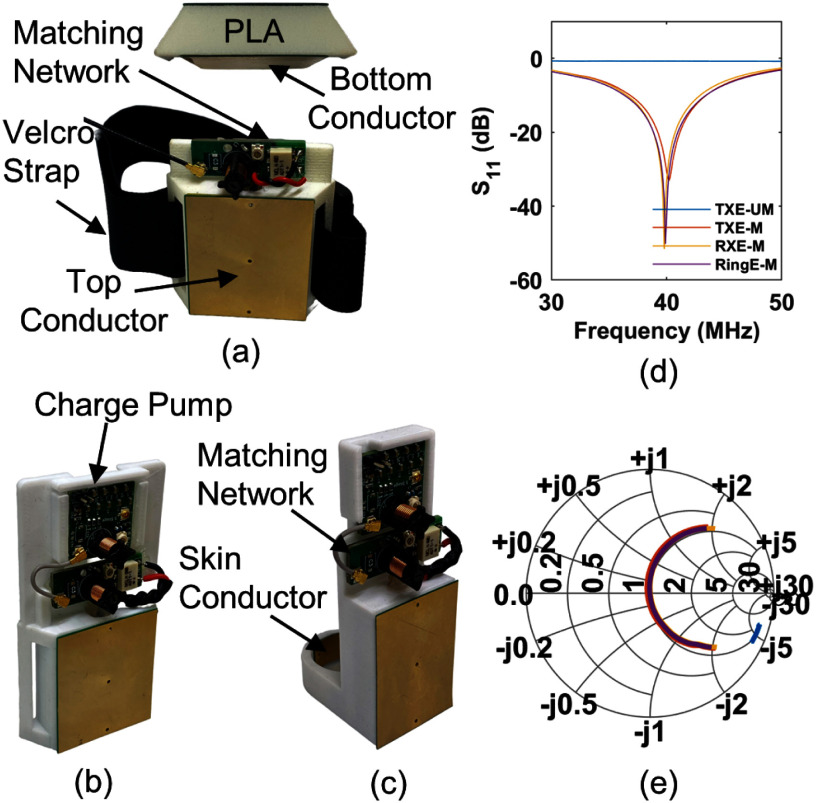
Electrode systems used in the (a) wrist, (b) leg and forearm, and (c) finger. (d) Measured $S_{11}$ responses, where TXE-UM is the unmatched transmitter (blue curve). TXE-M, RXE-M, and RingE-M are matched electrodes. (e) Impedance plots on a Smith chart.

Fig. 2(a) illustrates the transmitter electrode for limb placement. Fig. 2(b) and (c) show the corresponding receiver electrode configurations for limb and finger placement. The limb electrodes for both transmitter and receiver employ $\mathrm{30}\,\mathrm{mm} \times \mathrm{40}\,\mathrm{mm}$ skin and floating plates separated by $\mathrm{11}\,\mathrm{mm}$. The finger electrode uses a $\mathrm{20}\,\mathrm{mm} \times \mathrm{30}\,\mathrm{mm}$ floating plate and a $\mathrm{1}\,\mathrm{cm}$ wide skin conductor integrated into a $\mathrm{20.6}\,\mathrm{mm}$ diameter ring. All structures were fabricated using PLA plastic with a relative permittivity of 3.1.

Each electrode system incorporates a DC-DC isolated balun transformer and an impedance matching network. This eliminates capacitive coupling between the electrodes and the PCB ground, ensuring that the body and air paths form the dominant current loop. All electrodes are matched to $50\,\Omega$ using an LC network. Fig. 2(d) and 2(e) show the measured $S_{11}$ responses and the corresponding Smith chart plots.

#### 2) Transmitter System

**Fig. 3.**
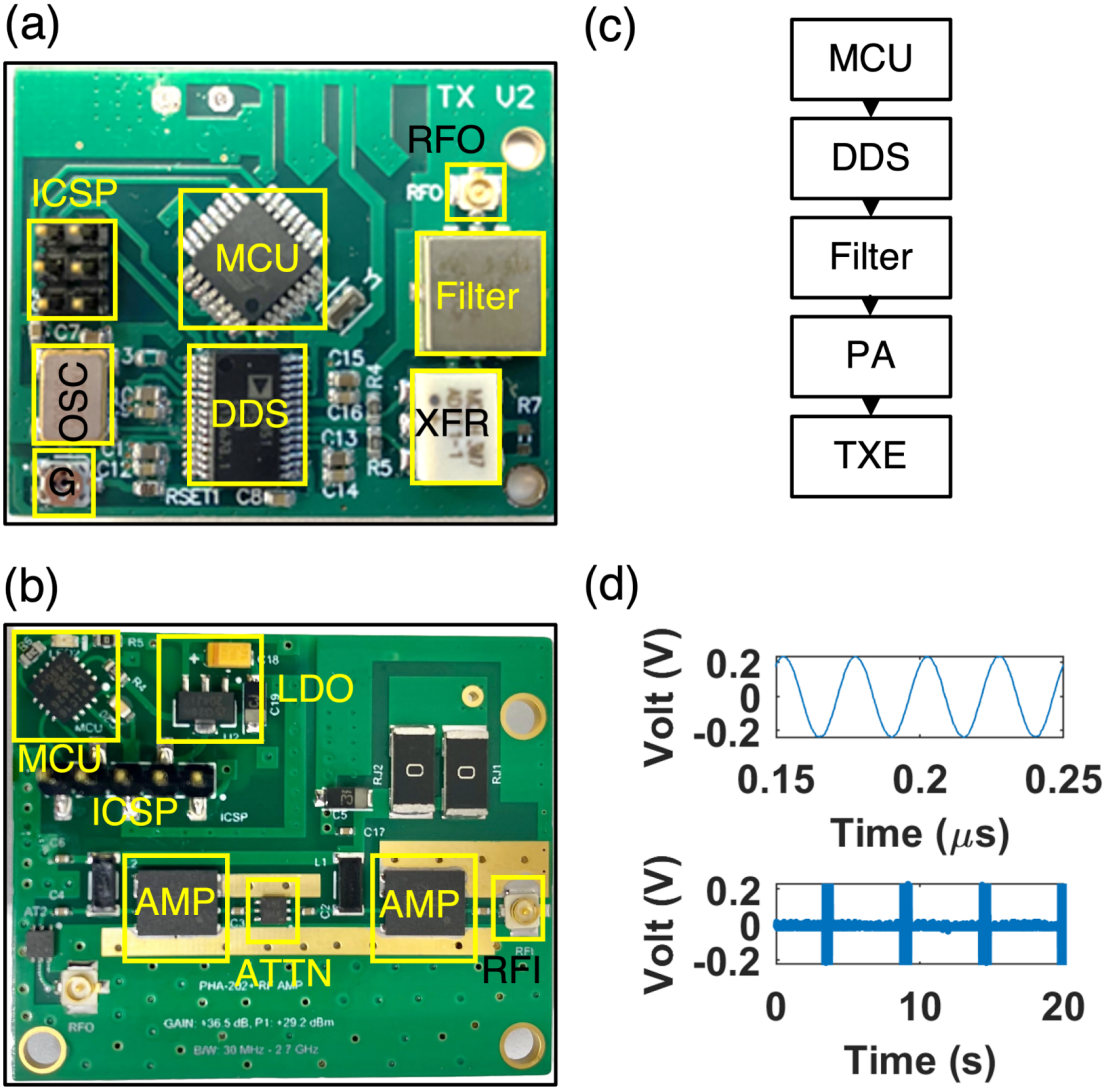
RF transmitter system. (a) RF generator PCB. (b) Power amplifier PCB. (c) Block diagram of the transmitter. (d) Pulsed 40 MHz waveform measured after a 30 dB attenuator.

The transmitter in the LCC platform provides a 40 MHz RF excitation signal. Fig. 3(a) and (b) show the PCB implementations of the RF generator and the power amplifier stages, which are powered by a 12 V battery. The RF generator uses a direct digital synthesizer (DDS; AD9851) controlled by a microcontroller (ATMEGA328). Fig. 3(c) summarizes the transmitter architecture.

To support energy harvesting in low-power embedded systems, two cascaded power amplifiers (PA; PHA202+) increase the DDS output to $\mathrm{27.5}\,\mathrm{dBm}$ RF peak power. The amplifier supply is duty cycled using an ultra-low-power microcontroller (PIC16LF8426) to reduce thermal dissipation. The RF duty cycle is 10 percent, corresponding to 500 ms on and 5 s off. The on-time allows the receiver capacitor to reach steady-state voltage, and the off-time allows both amplifier cooling and receiver capacitor discharge. Fig. 3(d) shows the resulting pulsed waveform after attenuation and termination.

#### 3) Power Receiver System

**Fig. 4.**
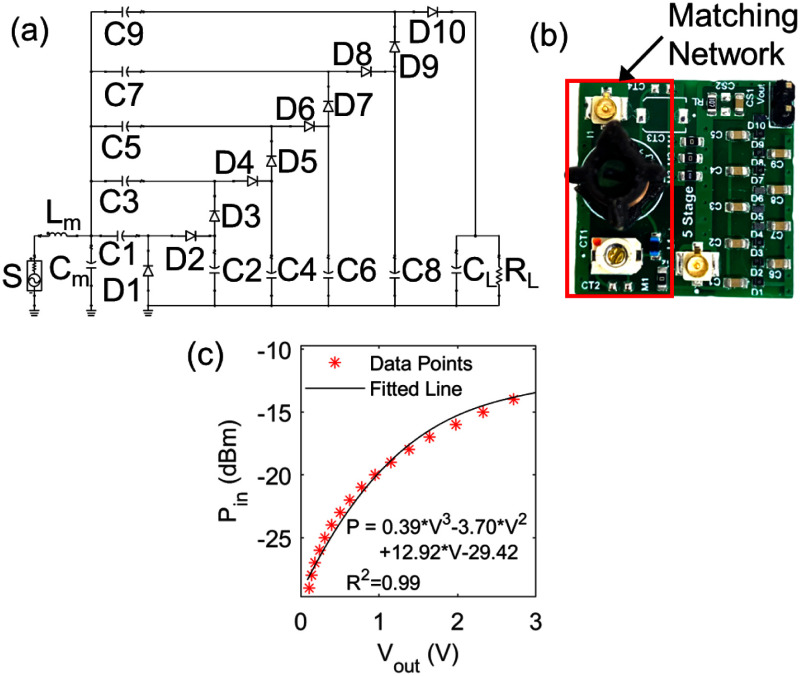
(a) Five-stage Dickson charge pump. (b) Receiver PCB implementation. (c) Peak RF input power calibration curve.

The received RF signal is rectified and boosted using a five-stage Dickson charge pump, which stores output energy on a capacitor. Diodes D1 through D9 are SMS7630 Schottky devices with approximately $\mathrm{180}\,\mathrm{mV}$ forward voltage, but these exhibit reverse leakage that increases with stage number. To reduce leakage in the final stage, we used SMS3922 for diode D10. The load consists of a parallel RC network with a $\mathrm{100}\,\mathrm{nF}$ capacitor and a $\mathrm{100}\,\mathrm{M}\Omega$ resistor, emulating the quiescent draw of an ultra-low-power embedded system. Fig. 4(b) shows the PCB layout of the receiver. Fig. 4(c) presents a calibration curve relating peak input RF power $P_{\mathrm{in}}$ to the steady-state DC output voltage $V_{\mathrm{out}}$. A third-order polynomial fit is used to estimate received RF power from measured DC voltage during experiments.

#### 4) Batteryless Ring Sensor

**Fig. 5.**
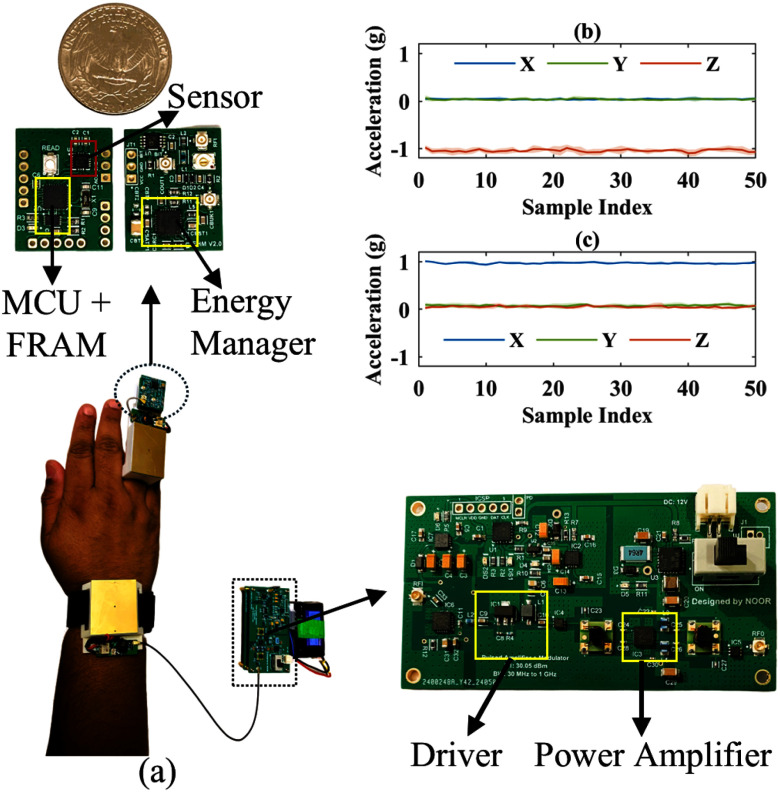
(a) Batteryless ring sensor with wrist-worn power transmitter. Accelerometer data captured when the hand is (b) neutral and (c) flexed.

Fig. 5(a) shows the batteryless ring sensor system, which serves as a benchmark application leveraging LCC, incorporating the previously described electrode, transmitter, and receiver designs. The sensor includes an ultra-low-power accelerometer (ADXL362), a microcontroller (STM32L432KCU6), and FRAM memory (MB85RC64TA) for offline data storage. Its firmware was optimized using dynamic clock scaling, peripheral clock gating, and deep sleep modes. The ADXL362 operated in low-power mode with a $ \pm 2g$ range and a sampling rate of $\mathrm{12.5}\,\mathrm{Hz}$. Samples were buffered in the internal FIFO and read in bursts via SPI to reduce microcontroller wakeups. A 250 ms sampling interval was maintained, and 50 samples were collected for evaluation.

### C. Modeling of LCC

Accurate characterization of LCC requires models that capture both the biophysical and electrical properties of the system. We developed a hybrid modeling framework that combines Finite Element Modeling (FEM) with lumped circuit analysis to evaluate channel gain, coupling capacitance, and three-dimensional electric field distributions. Modeling was conducted using ANSYS HFSS and Keysight ADS.

#### 1) Biophysical FEM Model

**Fig. 6.**
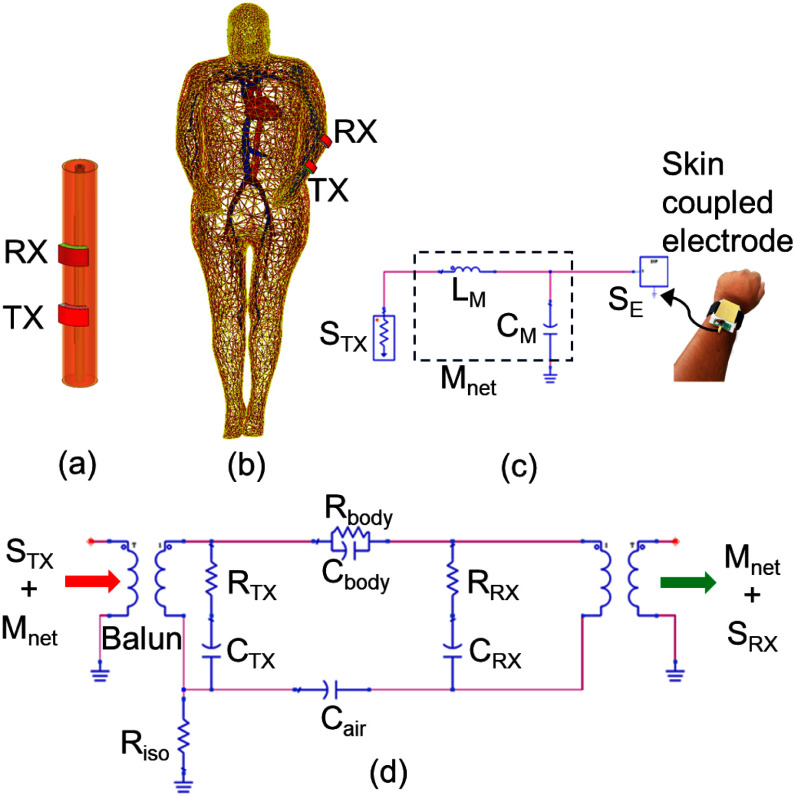
(a) Multilayer cylindrical FEM forearm model. (b) NEVA hand FEM model for a 10 cm channel. (c) LC matching for limb electrodes. (d) Equivalent circuit model of the IBPT system.

We developed a multilayer cylindrical FEM model of a human forearm (Fig. 6(a)) consisting of skin, fat, muscle, cortical bone, and bone marrow layers [27]. Tissue electrical properties were taken from Gabriel et al. [28] and FCC data [29]. The electrodes were modeled as $\mathrm{30}\,\mathrm{mm} \times \mathrm{40}\,\mathrm{mm}$ differential pairs embedded in PLA with $\mathrm{11}\,\mathrm{mm}$ spacing. Absorbing boundary conditions were applied, and two-port $S$-parameters were solved under matched impedances.

We also used the NEVA full-scale human model [26] (Fig. 6(b)) to analyze electric field distribution for a 10 cm hand channel. Simulations were performed without external ground references so that only inter-device coupling contributed to the resulting field and path gain.

#### 2) Circuit Model

A complementary circuit model was developed using measured electrode impedances. Fig. 6(c) shows an LC matching network with $L_{m}=894\,\mathrm{nH}$ and $C_{m}=2.3\,\mathrm{pF}$ connected to a $50\,\Omega$ source. Fig. 6(d) depicts the full equivalent LCC circuit, where electrode impedances are represented using lumped parameters $R_{TX}$, $C_{TX}$, $R_{RX}$, and $C_{RX}$. Based on prior work, a 10 cm limb channel can be approximated with $R_{body}=65\,\Omega$ and $C_{body}=25\,\mathrm{pF}$ [17]. Path gain is dominated by the air path impedance set by $C_{air}$, which is substantially larger than the body path impedance. A $\mathrm{100}\,\mathrm{G}\Omega$ isolation resistor $R_{iso}$ was added to satisfy ADS requirements for a closed DC loop.

## III. Experimental Setup

We conducted a series of experiments involving human subjects to analyze channel gain across multiple body channels during both stationary conditions and dynamic movements. Ten male participants, aged 25 to 35 with body mass indices (BMI) ranging from 25 to 43, were recruited as a convenience sample consisting of students, faculty, and staff within the engineering department. The Institutional Review Board at the University of Massachusetts Amherst approved the experimental protocol (IRB #2909), and all participants provided written informed consent outlining the potential risks and benefits. Experiments were performed in a laboratory environment with room temperatures between 19 and 27 $^{\circ }\mathrm{C}$ and humidity between 20 and 60 percent. For evaluation of the batteryless ring sensor (Fig. 5(a)), data were collected from a single subject because the device was developed in a later stage of the project following hardware optimization.

### A. Data Collection

**Fig. 7.**
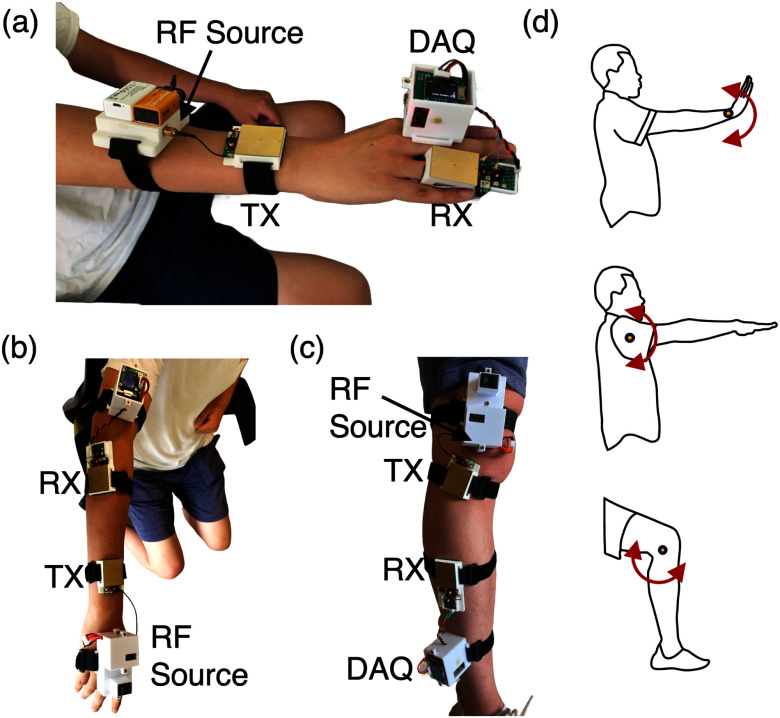
Experimental setups for (a) wrist-to-finger, (b) wrist-to-forearm, and (c) knee-to-ankle channel characterization using transmitter (TX) and receiver (RX) electrodes. (d) Motor tasks performed during dynamic actions.

We designed the experiments for LCC to establish a foundation for evaluating channel gain under realistic wearable conditions and to guide the development of our isolated data acquisition system described in Section III-B. Specifically, we considered three body channels: wrist-to-finger, wrist-to-forearm, and ankle-to-knee. Each configuration was tested under stationary conditions and dynamic motion to characterize how body part orientation, electrode alignment, and limb movement affect channel gain. Fig. 7(a), (b), and (c) illustrate the stationary configurations. Fig. 7(d) shows the dynamic motor tasks, which included wrist, shoulder, and knee flexion and extension performed in the sagittal plane for approximately 30 seconds. Participants were instructed to move through their full available range of motion, typically near $180^{\circ }$, although minor variation occurred among individuals.

In the wrist-to-finger configuration (Fig. 7(a)), the transmitter electrode was placed on the wrist and the receiver electrode on the ring finger (fourth proximal digit). The channel distance was maintained at approximately 11.5 cm. In the wrist-to-forearm configuration (Fig. 7(b)), the transmitter was placed on the wrist and the receiver on the forearm with a fixed channel length of 10 cm. We then reversed electrode positions to assess reciprocity and potential gain asymmetry. Additionally, for the configuration with the transmitter on the wrist and the receiver on the forearm, channel length was varied from 5 cm to 12 cm by adjusting the receiver electrode position. In the ankle-to-knee configuration (Fig. 7(c)), the transmitter was placed on the knee and the receiver on the ankle with a fixed separation of 10 cm.

The transmitter used continuous-wave carriers with a peak RF power of $\mathrm{18.5}\,\mathrm{dBm}$ for the wrist-to-finger measurements and pulsed carriers with a peak power of $\mathrm{27.5}\,\mathrm{dBm}$ at a 9.1 percent duty cycle for the wrist-to-forearm and ankle-to-knee channels. Continuous carriers at lower power were used for the wrist-to-finger configuration because this channel exhibited higher gain, and reduced power prevented saturation of the data acquisition device (see Section III-B). This also allowed evaluation of both continuous and duty-cycled power transfer modes.

For batteryless sensor evaluation, measurements were performed under two static hand postures: neutral and flexed positions. No hand movement occurred during data capture. Measurements were repeated five times for each posture to assess repeatability. Logged acceleration samples were retrieved from FRAM and transmitted via UART in the CSV format. During data transfer, the microcontroller temporarily increased its internal clock to 1 MHz to support UART communication at 9600 bps. Each acceleration sample consisted of comma-separated signed values for the X, Y, and Z axes for straightforward parsing during offline analysis.

### B. Data Acquisition System

**Fig. 8.**
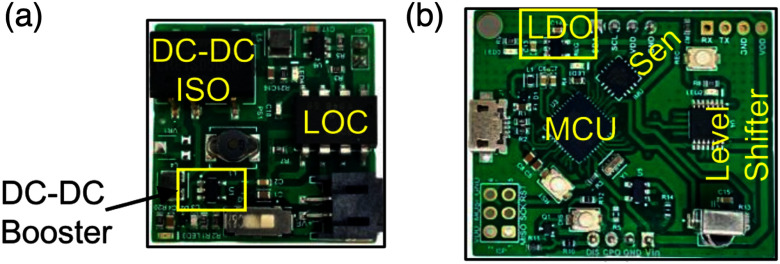
PCB implementation of (a) isolation hardware ($\mathrm{2.8}\,\mathrm{cm} \times \mathrm{2.8}\,\mathrm{cm}$) with a voltage booster and DC-DC power supply isolator for the linear optocoupler (LOC), and (b) the datalogger board ($\mathrm{3.4}\,\mathrm{cm} \times \mathrm{4.1}\,\mathrm{cm}$) including a voltage regulator (LDO), microcontroller (MCU), accelerometer (SEN), and SD card level shifter.

We developed a custom data acquisition (DAQ) system to ensure proper ground isolation so that measured channel gains accurately reflect conditions achievable in wearable systems rather than artifacts introduced by laboratory instrumentation. This design eliminates low-impedance return paths through test equipment and ensures that channel gain measurements represent realistic performance of the fully isolated LCC system. The isolation module (Fig. 8(a)) uses a linear optocoupler (LOC) to provide galvanic isolation between the data logger and the charge pump output. A separate opto-isolated switching circuit, controlled by a microcontroller (ATMEGA32U4), discharges the receiver's storage capacitor between measurements. The DAQ computes received power by counting the number of capacitor recharge cycles over a given time interval, and all data are stored on a removable SD card for offline processing. Fig. 8(b) shows the DAQ PCB implementation.

## IV. Results

### A. Path Gain Over Channel Lengths

**Fig. 9.**
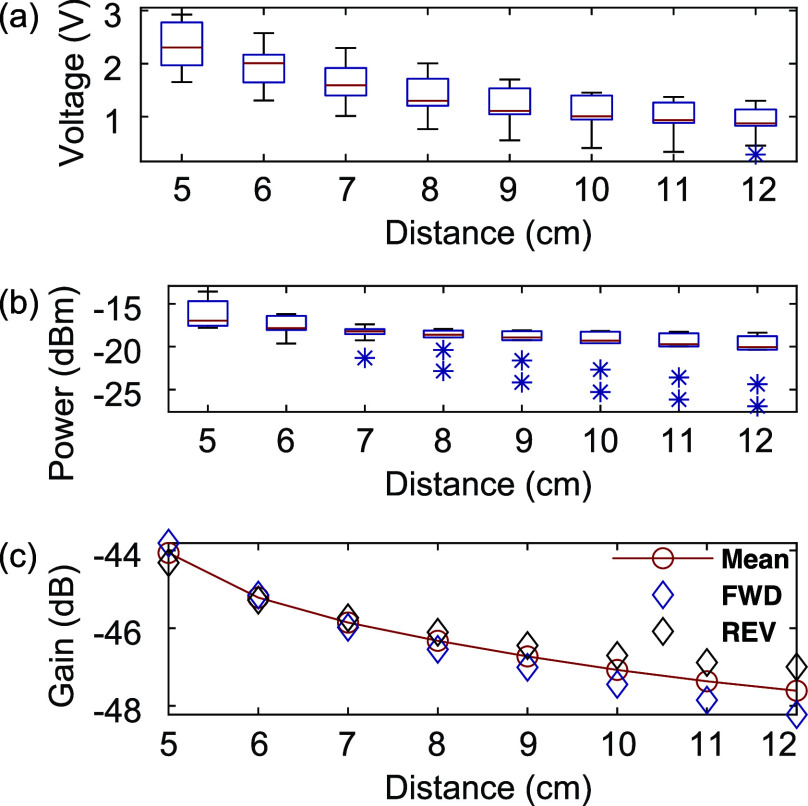
(a) Steady-state peak voltage from the charge pump. (b) Estimated received RF power. (c) Mean path gain for forward (FWD) and reverse (REV) measurements.

Fig. 9 summarizes the stationary upper-limb measurements collected while varying electrode separation from $\mathrm{5}\,\mathrm{cm}$ to $\mathrm{12}\,\mathrm{cm}$. Fig. 9(a) shows the steady-state output voltage produced by the charge pump at each distance. Using the calibration method from Fig. 4, the corresponding received RF power is estimated in Fig. 9(b). As expected, received power decreases gradually with distance, with an overall reduction of approximately $\mathrm{4}\,\mathrm{dB}$ across the measured range.

To examine channel reciprocity, path gain was measured in both forward and reverse configurations by swapping transmitter and receiver locations. Fig. 9(c) shows the resulting mean path gain across participants. Inter-body gain variation remained within $\mathrm{3}\,\mathrm{dB}$ for nearly all subjects, and forward and reverse measurements exhibited no meaningful differences. This supports the assumption that LCC behaves symmetrically over short channel lengths.

### B. FEM and Circuit Model Simulations

**Fig. 10.**
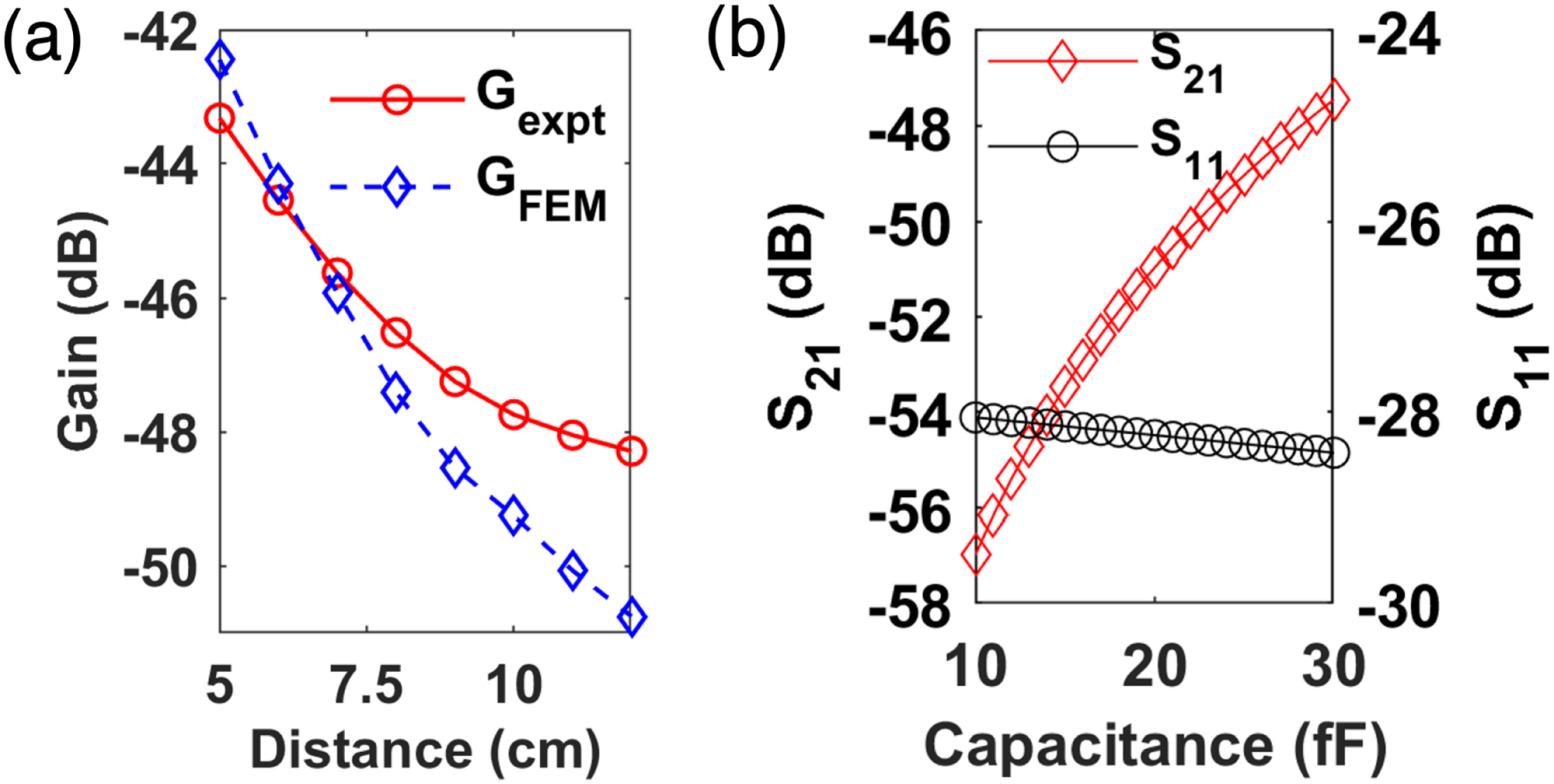
(a) Comparison of $G_{expt}$ and cylindrical FEM model ($G_{FEM}$). (b) Effect of coupling capacitance on $S_{21}$ and $S_{11}$.

Fig. 10(a) compares the simulated path gain from the cylindrical FEM model ($G_{FEM}$) with the empirical measurements ($G_{expt}$) across different channel lengths. The two results agree closely for separations up to $\mathrm{8}\,\mathrm{cm}$, and remain within $\mathrm{3}\,\mathrm{dB}$ of each other at longer distances. This consistency indicates that the FEM model captures the dominant coupling behavior of LCC and provides a reliable tool for exploring parameter variations.

The equivalent circuit model in Fig. 6(d) was used to examine how changes in the air-gap coupling capacitance influence path gain and impedance match. As shown in Fig. 10(b), varying the coupling capacitance from $\mathrm{10}\,\mathrm{fF}$ to $\mathrm{30}\,\mathrm{fF}$ produces a path gain change from approximately $-57\,\mathrm{dB}$ to $-47\,\mathrm{dB}$. These capacitance values fall within realistic ranges determined by electrode alignment, channel length, and local geometry. Across this sweep, return loss remains low, indicating that the matching network is effective and largely insensitive to small variations in coupling.

**Fig. 11.**
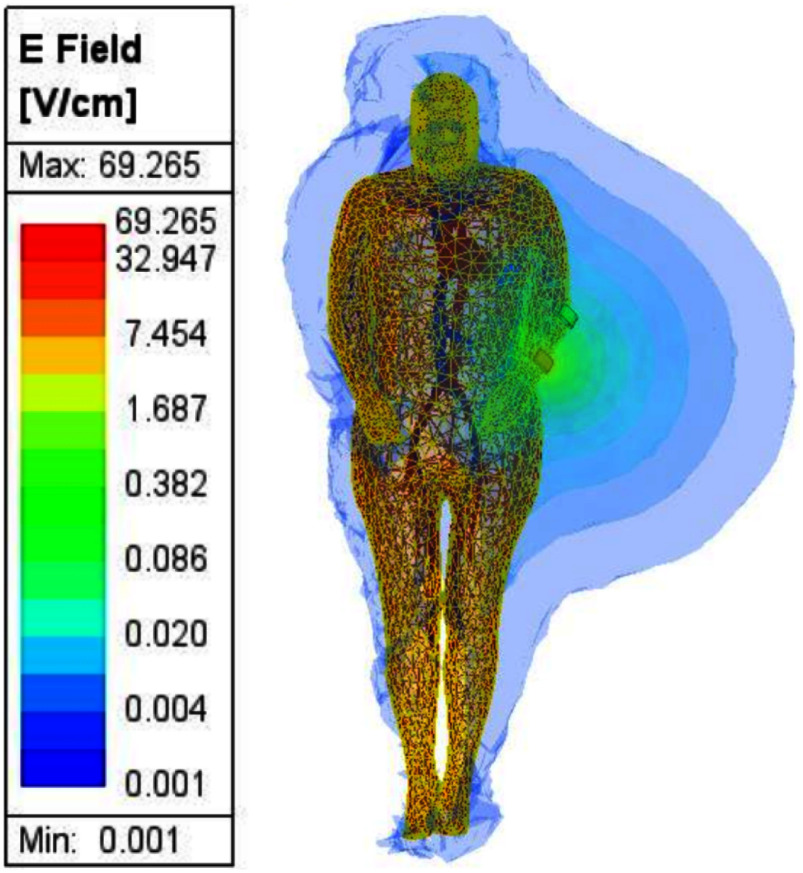
3D electric field distribution from the NEVA FEM model for a $\mathrm{10}\,\mathrm{cm}$ wrist-to-arm channel (fields $< 1\,\mathrm{mV/cm}$ omitted).

To visualize the field distribution, Fig. 11 presents the 3D electric field around the NEVA FEM model for a $\mathrm{10}\,\mathrm{cm}$ wrist-to-forearm channel. The field is concentrated around the electrode structures and decays rapidly within roughly one meter of the torso. This localized behavior aligns with the design goal of LCC, which relies on near-field capacitive effects rather than large-scale environmental coupling.

### C. Path Gain During Stationary Postures and Dynamic Actions

**Fig. 12.**
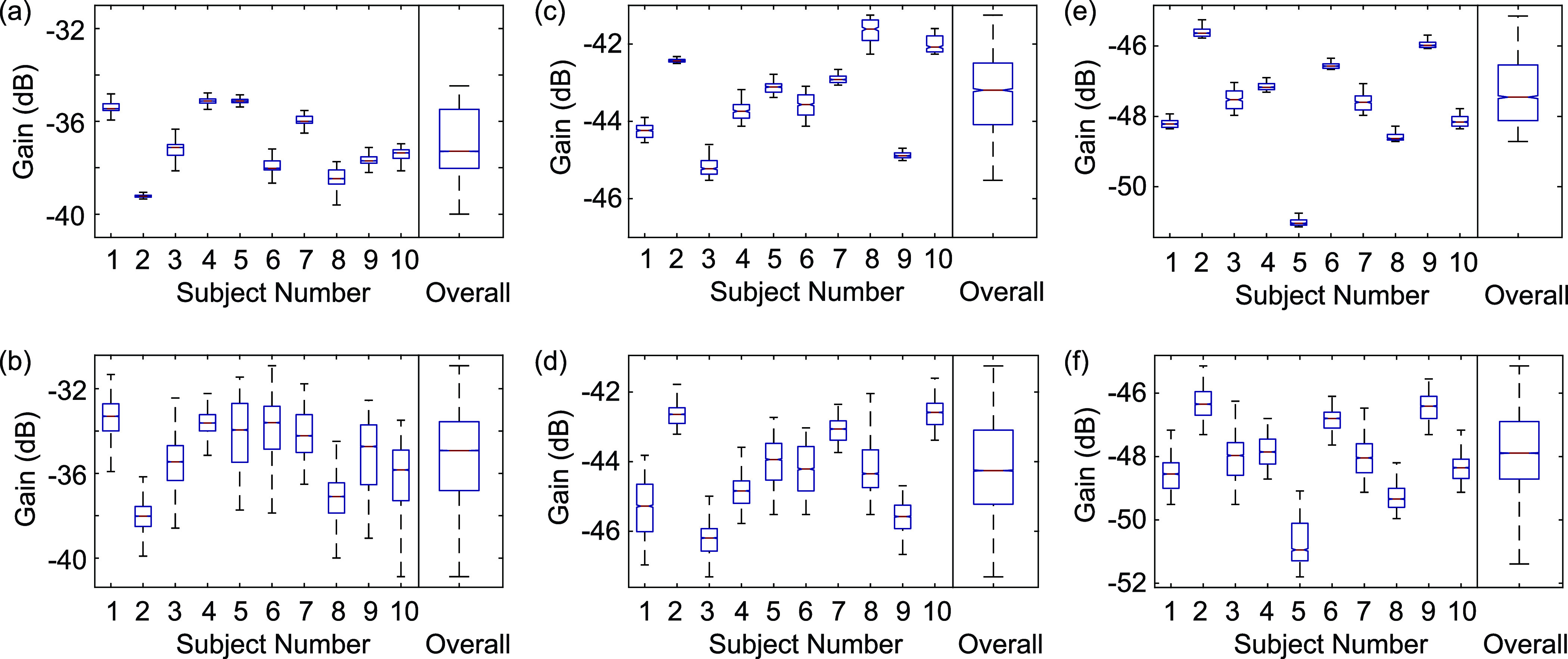
Path gains for (a) stationary finger, (b) moving finger, (c) stationary hand, (d) moving hand, (e) stationary leg, and (f) moving leg for $\mathrm{10}\,\mathrm{cm}$ channels across ten subjects.

Fig. 12 compares stationary and dynamic path gain measurements for the wrist-to-finger, wrist-to-forearm, and knee-to-ankle channels. These experiments quantify how motion impacts coupling when electrodes move through different orientations. For the wrist-to-forearm channel (Fig. 12(a), (b)), the mean stationary gain across participants was $-37\,\mathrm{dB}$, with individual values ranging from $-34.5$ to $-40\,\mathrm{dB}$. During wrist flexion and extension, mean gain increased slightly to $-35.2\,\mathrm{dB}$, although individual measurements spanned a broader range ($-42$ to $-30.5\,\mathrm{dB}$), reflecting transient changes in electrode orientation. For the wrist-to-finger channel (Fig. 12(c), (d)), mean stationary gain was $-43.4\,\mathrm{dB}$, shifting to $-44.2\,\mathrm{dB}$ during shoulder flexion and extension. The knee-to-ankle channel (Fig. 12(e), (f)) exhibited the lowest overall gain, with stationary values near $-47.5\,\mathrm{dB}$ and dynamic values near $-47.9\,\mathrm{dB}$.

Across all channels, intra-subject variation during stationary conditions was small (approximately $\mathrm{1}\,\mathrm{dB}$), indicating stable coupling when posture is fixed. During dynamic actions, intra-subject variation increased to approximately 11 to $\mathrm{12}\,\mathrm{dB}$, consistent with 3D electrode movement and brief deviations from optimal alignment.

### D. Ring Sensor Evaluation

The ring sensor evaluation demonstrates practical, batteryless operation of a wearable device powered by LCC. Fig. 5(b) shows accelerometer data during the neutral posture, where the Z-axis measures approximately $-1\,\mathrm{g}$ due to gravity, and the X and Y axes remain near zero. During the flexed posture (Fig. 5(c)), the sensor reorients such that the X-axis approaches $+1\,\mathrm{g}$. These changes are consistent across trials. The mean acceleration magnitudes were $\mathrm{1.0424}\,\mathrm{g} \pm 0.0258$ for the neutral posture and $\mathrm{0.9796}\,\mathrm{g} \pm 0.0132$ during flexion. These results confirm that the sensor reliably powers up, captures motion data, and distinguishes hand postures through gravitational alignment, illustrating the feasibility of batteryless sensing using LCC.

## V. Discussions

This paper introduces a localized capacitive coupling model for capacitively coupled IBPT and presents a comprehensive evaluation supported by empirical measurements and simulation analyses. Our results demonstrate mean path gains ranging from $-48$ to $-43\,\mathrm{dB}$ for the wrist to forearm and knee to ankle channels at $\mathrm{10}\,\mathrm{cm}$, while the wrist to finger channel at $11.5 \pm \mathrm{1.2}\,\mathrm{cm}$ achieves higher gains between $-37$ and $-35\,\mathrm{dB}$.

The higher gain observed in the wrist to finger channel is likely due to the smaller skin surface area involved compared to the wrist to forearm and knee to ankle channels. When a floating conductor is positioned parallel to the skin, the skin's conductivity creates strong localized capacitive coupling that reduces the contribution of the distant floating conductor. A smaller skin area increases the proportion of fringing field directed toward the receiving electrode, which enhances the inter-device coupling capacitance and produces higher gain for this channel.

Body movements significantly increased gain variation while having minimal effect on the median gain across participants, as shown in Fig. 12. This behavior is expected because small changes in proximity and orientation of the floating electrodes alter the air path capacitance on the order of femtofarads. These changes strongly influence the inter-device coupling and therefore the instantaneous gain. The wrist to forearm and knee to ankle channels exhibited smaller gain variations during movement than the wrist to finger channel. This is attributed to the fact that the electrode separation remained nearly constant during wrist or knee flexion, while finger motion changed both proximity and orientation of the electrodes.

Impedance matching also plays an important role in maximizing path gain. We observed inter-subject mean gain variations of up to $\mathrm{5}\,\mathrm{dB}$ in Fig. 12(a), (c), and (e). However, once the electrode match reaches a reasonable threshold (approximately less than $-20\,\mathrm{dB}$), further improvements in skin electrode matching have limited impact. Even with a relatively high skin resistance, the effective body impedance is reduced by the shunt capacitance of the body path. As a result, the overall loop gain is dominated by the air path coupling capacitance. Improving gain therefore requires enhancements to fringing field coupling or increases in carrier power. Carrier power, however, must comply with human safety constraints such as the $4\,\mathrm{W/kg}$ specific absorption rate limit for limbs [30]. To address this constraint, our system used pulsed carrier transmission with a 9.1 percent duty cycle to reduce average absorption.

Beyond single-link characterization, the localized field patterns in Fig. 11 suggest important system-level tradeoffs for future multi-node networks. The strong confinement of the electric field near the electrodes is advantageous for limiting unintended exposure and reducing interference with nearby devices, but it also implies that a single transmitter cannot efficiently power sensors distributed over the entire body. Supporting larger networks may therefore require multiple coordinated transmitters, duty-cycled scheduling of power transfer across subsets of nodes, or opportunistic use of environmental conductors in specific settings. These approaches would increase system complexity, but they also create opportunities for application-specific optimization, for example by prioritizing critical sensors or leveraging information about user posture and activity to allocate power.

Several limitations of this study merit discussion. First, the relatively small sample size may limit generalizability. Participants were recruited from within an engineering department, and broader demographic diversity would strengthen future validation of intra-body gain characteristics. Second, all study participants were male. Although tissue electrical properties can vary across genders, the dominant factors for path gain in LCC arise from air path impedance and coupling capacitance, which reduces concerns about gender-specific effects. Nevertheless, variations in subcutaneous tissue thickness, hydration, and body composition may influence effective coupling capacitance and should be systematically evaluated in future studies including both male and female participants across a wider BMI distribution. Third, we examined a limited set of body movements. While these movements captured a broad range of electrode orientations and channel configurations, they do not represent the full spectrum of daily human motion. Additional studies that include more complex activities would improve generalizability. Lastly, all experiments were conducted in a controlled laboratory environment. Future work should investigate path gain in real-world settings that include environmental perturbations, clothing, sweating, and longer-term wear.

## VI. Conclusion

This work investigated intra-body power transfer using the LCC method across multiple body locations. We showed that an isolated, impedance-matched, differential, and wearable electrode system with a $\mathrm{10}\,\mathrm{cm}$ separation consistently achieves a mean path gain of $-46 \pm \mathrm{3}\,\mathrm{dB}$ at the hand and leg, with limited sensitivity to inter-subject variation. By combining empirical measurements with FEM and circuit models, we provided design insights into how coupling capacitance, electrode geometry, isolation, and matching influence power transfer in short-channel IBPT systems. Our findings confirm that localized capacitive coupling is a viable mechanism for batteryless power delivery over short distances on the human body. They also highlight the sensitivity of coupling to electrode geometry, air-path capacitance, and movement patternsfactors that inform the design of future wearable sensor systems based on LCC. At publication, we will open source our hardware designs and simulation configurations and make them publicly available to the research community to facilitate further development of batteryless wearable sensing platforms.

## References

[1] N. Mohammed, R. Wang, R. W. Jackson, Y. Noh, J. Gummeson, and S. I. Lee, ShaZam: Charge-free wearable devices via intra-body power transfer from everyday objects, in Proc. ACM Interactive, Mobile, Wearable Ubiquitous Technol., vol. 5, no. 2, pp. 1 25, 2021.

[2] R. Shukla, N. Kiran, R. Wang, J. Gummeson, and S. I. Lee, SkinnyPower: Enabling batteryless wearable sensors via intra-body power transfer, in Proc. 17th Conf. Embedded Networked Sensor Syst., 2019, pp. 68 82.

[3] A. P. Sample, D. J. Yeager, P. S. Powledge, A. V. Mamishev, and J. R. Smith, Design of an RFID-based battery-free programmable sensing platform, IEEE Trans. Instrum. Meas., vol. 57, no. 11, pp. 2608 2615, Nov. 2008.

[4] V. Liu, A. Parks, V. Talla, S. Gollakota, D. Wetherall, and J. R. Smith, Ambient backscatter: Wireless communication out of thin air, ACM SIGCOMM Comput. Commun. Rev., vol. 43, no. 4, pp. 39 50, 2013.

[5] N. Mohammed, R. W. Jackson, J. Gummeson, and S. I. Lee, Wireless intra-body power transfer via capacitively coupled link, in Proc. IEEE-EMBS Int. Conf. Wearable Implantable Body Sensor Netw., 2022, pp. 1 4.

[6] J. Li, Y. Dong, J. H. Park, and J. Yoo, Body-coupled power transmission and energy harvesting, Nature Electron., vol. 4, no. 7, pp. 530 538, 2021.

[7] Y. Dong et al., Body-coupled power transceiver with node-specific body-area powering, in Proc. ESSCIRC 2021-IEEE 47th Eur. Solid State Circuits Conf., 2021, pp. 423 426.

[8] N. Modak et al., EQS Res-HBC: A 65-nm electro-quasistatic resonant 5240 $\mu$w human whole-body powering and 2.19 $\mu$w communication SoC with automatic maximum resonant power tracking, IEEE J. Solid-State Circuits, vol. 57, no. 3, pp. 831 844, Mar. 2022.

[9] D. Yang, S. Maity, and S. Sen, Physically secure wearablewearable through-body interhuman body communication, Front. Electron., vol. 2, 2022, Art. no. 807051.

[10] J. Park, H. Garudadri, and P. P. Mercier, Channel modeling of miniaturized battery-powered capacitive human body communication systems, IEEE Trans. Biomed. Eng., vol. 64, no. 2, pp. 452 462, Feb. 2017. 27164566 10.1109/TBME.2016.2560881

[11] N. Modak, M. Nath, B. Chatterjee, S. Maity, and S. Sen, Bio-physical modeling of Galvanic human body communication in electro-quasistatic regime, IEEE Trans. Biomed. Eng., vol. 69, no. 12, pp. 3717 3727, Dec. 2022. 35594211 10.1109/TBME.2022.3176541

[12] E. Wen, D. F. Sievenpiper, and P. P. Mercier, Channel characterization of magnetic human body communication, IEEE Trans. Biomed. Eng., vol. 69, no. 2, pp. 569 579, Feb. 2022. 34347590 10.1109/TBME.2021.3101766

[13] M. Nath, A. K. Ulvog, S. Weigand, and S. Sen, Understanding the role of magnetic and magneto-quasistatic fields in human body communication, IEEE Trans. Biomed. Eng., vol. 69, no. 12, pp. 3635 3644, Dec. 2022. 35560087 10.1109/TBME.2022.3174959

[14] T. G. Zimmerman, Personal area networks: Near-field intrabody communication, IBM Syst. J., vol. 35, no. 3.4, pp. 609 617, 1996.

[15] A. Datta, M. Nath, D. Yang, and S. Sen, Advanced biophysical model to capture channel variability for EQS capacitive HBC, IEEE Trans. Biomed. Eng., vol. 68, no. 11, pp. 3435 3446, Nov. 2021. 33872142 10.1109/TBME.2021.3074138

[16] J. Yoo, Body area network: Connecting and powering things together around the human body, IEEE Solid-State Circuits Mag., vol. 15, no. 2, pp. 49 58, Spring 2023.

[17] R. Xu, H. Zhu, and J. Yuan, Electric-field intrabody communication channel modeling with finite-element method, IEEE Trans. Biomed. Eng., vol. 58, no. 3, pp. 705 712, Mar. 2011. 21095853 10.1109/TBME.2010.2093933

[18] A. Kong, D. Kim, and C. Harrison, Power-over-skin: Full-body wearables powered by intra-body RF energy, in Proc. 37th Annu. ACM Symp. User Interface Softw. Technol., 2024, pp. 1 13.

[19] H. Cho, J.-H. Suh, C. Kim, S. Ha, and M. Je, An intra-body power transfer system with $>$1-mW power delivered to the load and 3.3-V DC output at 160-cm of on-body distance, IEEE Trans. Biomed. Circuits Syst., vol. 16, no. 5, pp. 852 866, Oct. 2022. 35895660 10.1109/TBCAS.2022.3194278

[20] J. Sakai, L.-S. Wu, H.-C. Sun, and Y.-X. Guo, Balun's effect on the measurement of transmission characteristics for intrabody communication channel, in Proc. IEEE MTT-S Int. Microw. Workshop Ser. RF Wireless Technol. Biomed. Healthcare Appl., 2013, pp. 1 3.

[21] M. D. Pereira, G. A. Alvarez-Botero, and F. R. de Sousa, Characterization and modeling of the capacitive HBC channel, IEEE Trans. Instrum. Meas., vol. 64, no. 10, pp. 2626 2635, Oct. 2015.

[22] G. A. lvarez-Botero, Y. K. Hernndez-Gmez, C. E. Tellz, and J. F. Coronel, Human body communication: Channel characterization issues, IEEE Instrum. Meas. Mag., vol. 22, no. 5, pp. 48 53, Oct. 2019.

[23] J. Bae, H. Cho, K. Song, H. Lee, and H.-J. Yoo, The signal transmission mechanism on the surface of human body for body channel communication, IEEE Trans. Microw. Theory Techn., vol. 60, no. 3, pp. 582 593, Mar. 2012.

[24] F. Maamir, M. Guiatni, S. Tedjini, S. Gaoua, R. Touhami, and F. Abed, Optocoupler effect on in-vivo measurements characterization for human body communication, in Proc. 18th Mediterranean Microw. Symp., 2018, pp. 345 348.

[25] V. Semiconductors, Linear optocoupler, high gain stability, wide bandwidth, A A, vol. 100, no. 2, 2015, Art. no. 3.

[26] S. N. Makarov et al., Virtual human models for electromagnetic studies and their applications, IEEE Rev. Biomed. Eng., vol. 10, pp. 95 121, 2017. 28682265 10.1109/RBME.2017.2722420 PMC10502908

[27] J. Mao, H. Yang, Y. Lian, and B. Zhao, A self-adaptive capacitive compensation technique for body channel communication, IEEE Trans. Biomed. Circuits Syst., vol. 11, no. 5, pp. 1001 1012, Oct. 2017. 28644812 10.1109/TBCAS.2017.2695058

[28] S. Gabriel, R. Lau, and C. Gabriel, The dielectric properties of biological tissues: III. parametric models for the dielectric spectrum of tissues, Phys. Med. Biol., vol. 41, no. 11, 1996, Art. no. 2271. 10.1088/0031-9155/41/11/003 8938026

[29] Body tissue dielectric parameters, Sep. 2020. [Online]. Available: https://www.fcc.gov/general/body-tissue-dielectric-parameters

[30] International commission on non-ionizing radiation protection, Guidelines for limiting exposure to time-varying electric and magnetic fields (1 Hz to 100 kHz), Health Phys., vol. 99, no. 6, pp. 818 836, 2010. 21068601 10.1097/HP.0b013e3181f06c86

